# Sorption and Textural Properties of Activated Carbon Derived from Charred Beech Wood

**DOI:** 10.3390/molecules26247604

**Published:** 2021-12-15

**Authors:** Michal Zgrzebnicki, Agnieszka Kałamaga, Rafal Wrobel

**Affiliations:** Department of Catalytic and Sorbent Materials Engineering, Faculty of Chemical Technology and Engineering, West Pomeranian University of Technology, Szczecin, Piastów Ave. 42, 71-065 Szczecin, Poland; michal.zgrzebnicki@zut.edu.pl (M.Z.); agnieszka.kalamaga@zut.edu.pl (A.K.)

**Keywords:** activated carbon, sorption capacity, carbon dioxide, ethene, butane

## Abstract

The aim of this study was to prepare activated carbon materials with different porous structures. For this purpose, the biomass precursor, beech wood, was carbonized in an inert atmosphere, and the obtained charcoal was physically activated using carbon dioxide at 1273 K. Different porous structures were obtained by controlling the time of the activation process. Prepared materials were characterized in terms of textural (N_2_ sorption at 77 K), structural (XRD), and sorption properties (CO_2_, C_2_H_4_, C_4_H_10_). The shortest activation time resulted in a mostly microporous structure, which provided a high sorption of CO_2_. Increasing the activation time led to an increasing of the pores’ diameters. Therefore, the highest ethene uptake was obtained for the material with an intermediate activation time, while the highest butane uptake was obtained for the material with the highest activation time.

## 1. Introduction

Nowadays, much attention is being paid to environmental issues. More strict emission limits are being established in order to mitigate the negative influence on the surrounding environment. Moreover, some gases might be simply redundant and negatively affect some human activities. For instance, the following gases could be taken into consideration: carbon dioxide, ethene, and butane.

Carbon dioxide is known as a greenhouse gas. It absorbs infrared radiation emitted from the heated surface of Earth, which results in an increasing average temperature [1]. It was estimated that before the industrial revolution, the concentration of CO_2_ was roughly 280 ppm, while in 2021, it reached 415 ppm. Any further increase in CO_2_ concentration might lead to a further temperature increase, which might lead to dangerous weather phenomena. In order to prevent further climate warming, methods of reducing CO_2_ emission are being researched, including adsorption on solid sorbents.

Ethene is a commonly used material in the polymer production [2]. Moreover, it is emitted by some climacteric plants, such as tomatoes [3,4,5,6,7,8,9,10]. The presence of ethene accelerates the fruit maturation. Therefore, ethene is a crucial gas in the food storage, and methods of controlling the packing atmosphere are necessary.

While the adsorption of CO_2_ and C_2_H_4_ at 303 K, 1 atm requires micropores up to 0.7 nm and 1.0 nm, respectively [11], butane adsorption requires wider pores [12,13]. Therefore, it can be used as a model gas in order to test the adsorption capacity for some similar molecules. Moreover, butane is commonly used as a fuel for lighters or burners; thus, its removal from residual gas might be considered as necessary. Moreover, butane might be useful for the development of sorbents used in the prevention of breathing effect in gasoline tanks in cars. This effect leads to a loss of gasoline through evaporation in regions with high-temperature fluctuations between day and night.

In general, the emission of gaseous pollution might be reduced by using solid sorbents. In comparison with absorption using liquids, it requires less heat to regenerate the adsorbent [14]. Moreover, the adsorption process does not include dangerous chemicals, such as liquid amines in absorption. Suitable material for the adsorption of carbon dioxide, ethene, or butane is activated carbon.

An activated carbon (AC) is a material with a well-developed porous structure, which results in high specific surface area and very good sorption properties. The most common applications are a catalyst support [15,16], carbon electrodes [17], and an adsorbent used for the removal of undesirable compounds from gas [18] or liquid phase [19]. AC is obtained by the carbonization of carbonaceous material at temperatures above 723 K in an inert atmosphere, which is followed by an activation step at temperatures above 973–1073 K in an atmosphere depending on the type of process—atmosphere containing water vapor and/or carbon dioxide for physical type [20,21,22] and an inert for chemical type but with the presence of some additional compounds, such as KOH [23,24] or H_3_PO_4_ [25,26]. This particular step tailors the final sorption properties of AC.

The most common raw materials for the production of activated carbon are bituminous coal [27], lignite [28,29], peat [30], and biomass, which contains among other materials coconut shells [31,32], fruit stones [33], and wood [34,35,36]. Depending on the starting material, final AC might contain some amount of inorganic impurities, which after burning the carbon matrix creates ash. The removal of those impurities, even from the final product, might lead to an enhancement of sorption properties [37]. It can be performed by acid or alkali treatment, which can dissolve compounds such as alumina, iron oxide, silica, and common carbonates. Therefore, to reduce the production cost of activated carbon, other starting materials should be used rather than bituminous coal, lignite, and peat. For instance, a more suitable starting material is biomass. It is a relatively cheap, renewable resource, which is compatible with the environment. Moreover, it does not contain heavy elements; thus, activated carbon derived from biomass might find another application in pharmacology and cosmetics. Nevertheless, the price of biomass might vary significantly depending on its source. For instance, the use of fruit stones and leaves might lead to a considerable cost increase and might even encounter issues with the availability of the raw material. The solution of this problem is the use of wood.

Several papers concerning activated carbons produced from wood have been published previously [34]. Demiya et al. obtained activated carbon from rubber wood sawdust by CO_2_ activation at 1013 K for 1 h [36]. The specific surface area (SSA) and pore volume reached 465 m^2^/g and 0.239 cm^3^/g, respectively. Yusop et al. used acacia wood as a precursor to obtain activated carbon [38]. Samples were prepared via physicochemical activation. At first, they were impregnated with KOH followed by CO_2_ gasification under microwave heating. As a result, the specific surface area and average pore diameter reached 1045 m^2^/g and 2.78 nm, adequately. Gómez-Serrano et al. carried out the chemical activation of walnut wood by 36 wt % H_3_PO_4_ treatment [39]. The activation process was conducted at 723 K for 4 h. SSA was determined by the BET method using adsorption/desorption isotherms of N_2_ at 77 K and amounted to 769 m^2^/g.

Beech is a tree species growing on the plains and in the hills and lower mountain ranges [40,41]. It is mainly concentrated in central and western Europe (north Poland, north Germany, south Scandinavia, north France, and south England). For this reason, the price of beech wood is relatively low. The average price (the lowest quality class; 1-thickness class—middle diameter of log < 24 cm) in Poland reaches 50 Euro/m^3^. In comparison, the prices of ash and oak wood are 65 Euro/m^3^ and 70 Euro/m^3^, respectively [42]. Beech wood is characterized by high hardness, wear resistance, strength, and bending capabilities. These features enable it to be applied in many sectors of industry e.g., boatbuilding, furniture, flooring, plywood, pulp, or musical instruments production [43,44,45]. In addition, beech wood represents important renewable energy source—firewood [46]. Pyrolysis of wood leads to the formation of biochar, bio-oil, and pyrolytic gases. Bio-oil and gases can find applications in heat and energy generation. Biochar is commonly used as fuel and in the production of activated carbon.

The aim of our study was to explain the influence of activation time on the textural, structural, and sorption properties of activated carbons derived from beech wood. Our research focused on measuring the sorption properties of obtained materials for three different gases. The following adsorbates were used: CO_2_, C_2_H_4_, and C_4_H_10_, and their kinetic diameters were 0.33 nm, 0.39 nm, and 0.42 nm, respectively. Samples were obtained through physical activation at 1273 K under CO_2_ atmosphere for different times. One of the samples was meant to start cooling down immediately after reaching 1273 K. It is expected that extending the activation time will lead to the development of new micro- and mesopores. Such an approach might be found useful for other researchers. Moreover, it should be pointed out that there are many scientific papers about activated carbons. Based on our study, 10–30% of these papers describe in general activated carbons from biomass. Searching the phrase ‘beech wood activated carbon’ results in a narrowed down range of papers, which is much below 1% of the initial activated carbon articles’ number. To the best of our knowledge, there is no research article about activated carbon derived from beech wood and characterized in terms of the textural, structural and, what is more important, sorption properties of CO_2_, C_2_H_4_, and C_4_H_10_ at 303 K and atmospheric pressure.

## 2. Materials and Methods

### 2.1. Materials

A batch of beech wood was carbonized in a tubular furnace under the flow of nitrogen at 773 K for 2 h. The obtained chunk of charcoal was crushed and milled until powder until a grain size <1 mm was obtained.

Physical activation was performed in the following manner: 1 g of charcoal powder was put into the ceramic boat and placed in a tubular furnace (STF 15/180, Carbolite Gero). A carbon dioxide flow of 15 mL/min was maintained through the whole process. The temperature was increased at a rate of 5 K/min up to the desired temperature of 1273 K, which was followed by cooling at a rate of 5 K/min. The activation level was controlled by the time of activation process at the desired temperature. The following activation times were applied: 0 min (cooling started immediately after reaching the desired temperature), 10 min, 30 min, and 60 min. The obtained samples were named using the scheme ACXX, where XX is the activation time in minutes, e.g., AC00 for the sample cooled immediately after reaching 1273 K. Charred beech wood, denoted as ‘AC’, was used as a reference material.

Nitrogen and carbon dioxide gases used during preparation were provided by Messer, and their purity was 5.0 and 4.5, respectively.

### 2.2. Methods

The porous structure of the obtained materials was characterized using volumetric adsorption of nitrogen at 77 K (Autosorb Instrument, Quantachrome). The specific surface area (SSA) was calculated using the Brunauer–Emmett–Teller (BET) equation. Based on the obtained data, the following pore volumes were evaluated: total pore volume (V_total_), mesopore volume (V_meso_), and micropore volume (V_micro_). Moreover, in order to obtain the pore size distribution (PSD), density functional theory (DFT) for the slit-type pore model was applied to measured nitrogen isotherms.

Structural properties were obtained using the X-ray diffraction (XRD) technique. Measurements were performed on a diffractometer (Empyrean, PANalytical) equipped with a wide-angle detector (PIXcel 3D, PANalytical) and a monochromator, which lowered the signal to noise ratio. Measurements were performed in a 2θ range of 10–90°, using copper radiation (K_α1_ = 0.154056 nm). The interpretation of obtained diffractograms was performed using HighScore Plus software (Malvern PANalytical).

Sorption properties were measured using thermogravimetric analysis (TGA). The mass of activated carbons prepared for TGA analysis was in the range of 0.1 to 0.3 g. Measurements of CO_2_, C_2_H_4_, and C_4_H_10_ uptakes were performed under a constant flow of 40 cm^3^/g particular adsorbate. The thermobalance operated under atmospheric pressure. Measurements were performed in following manner—heating up to the desired temperature with a rate of 10 K/min, stabilization for 10 min, and cooling to 303 K. The desired temperature for carbon dioxide and ethene measurements was 523 K, while for butane, it was 573 K. A higher temperature for butane was required due to insufficient sorbent regeneration at 523 K.

## 3. Results and Discussion

### 3.1. Textural Results

**Figure 1:**
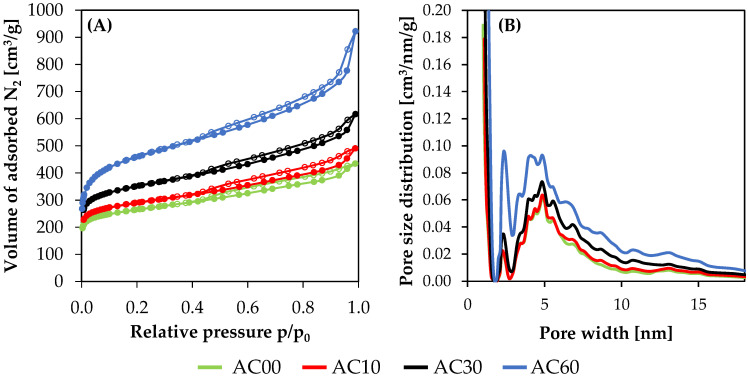
Results of N_2_ volumetric sorption at 77 K: (**A**) isotherms and (**B**) pore size distributions. Adsorption and desorption isotherms were indicated by filled and empty points, respectively.

The obtained nitrogen adsorption/desorption isotherms at 77 K, as well as pore size distributions, are presented in the Figure 1. Based on the obtained data, textural parameters were calculated and are presented in Table 1. The results of nitrogen adsorption at cryogenic temperatures provided general information about the micropores and more detailed information about the narrow mesopores.

Based on classification established by IUPAC, the obtained isotherms (Figure 1A) exhibited type IV(a), which is characteristic for mesoporous materials [47]. The distinctive feature of this particular type is the presence of hysteresis phenomena on adsorption/desorption isotherms. This hysteresis indicates that materials contain pores with diameters above 4 nm.

The PSD presented in the Figure 1B was presented for diameters up to 18 nm, due to the negligible amount of pores above this diameter. The presented results clearly indicate that extending the time of activation leads to increasing the amount of mesopores. It might be a combined result of merging nearby pores and simple widening of the pores. Moreover, it might be seen in Figure 1B that during activation, either the amount or diameter of the mesopores increases.

Table 1 presents the textural properties of the obtained materials. It is clearly visible that the specific surface area for the obtained materials is increasing with the activation time. However, the more developed the porous structure, the less the yield after the activation process. Moreover, it is worth mentioning that material AC00, which was activated through heating up to 1273 K and immediate cooling, resulted in a decent development of SSA equal to 990 m^2^/g. On the other hand, the highest SSA was obtained for material AC60 with the highest activation level. Furthermore, the applied physical activation procedure did not develop purely microporous activated carbons but rather materials with either micropores and mesopores. One can notice that all of the provided textural parameters increase with the increasing time of the activation process. Therefore, material AC00 with the shortest time of activation revealed the lowest content of micropores and mesopores. On the other hand, material AC60 with the longest time of activation revealed the highest amount of micropores and mesopores.

Additional results for materials obtained in other studies were introduced in Table 1 for comparison purposes. The provided materials were chosen based on the highest value of the specific surface area calculated from the BET equation. As it might be seen, the physical activation of beech wood under proper conditions results in significant development of the porous structure, which leads to high values of S_BET_. In fact, the S_BET_ value for material AC60 is the third highest value in Table 1. This means that there are materials with better porous properties, but these activated carbons might be obtained from exotic materials such as coconut shell, whose availability is regionally limited. On the other hand, beech wood might be a good resource for the production of activated carbons in central and eastern Europe.

Moreover, it is well-known that the higher the value of S_BET_, the lesser the yield of the process. It is strongly affected by the chemical composition of the starting material, i.e., the content of lignocellulose polymers. However, in case of some polymers, such as in this example PET, a higher yield after the activation process might be obtained.

### 3.2. Sorption Results

**Figure 2:**
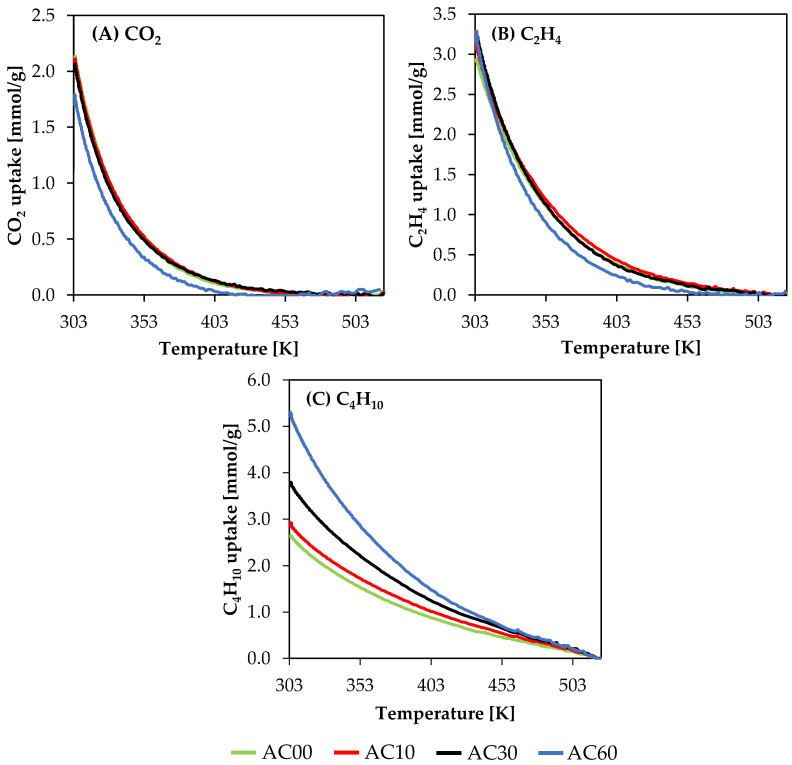
Adsorption isobars: (**A**) carbon dioxide, (**B**) ethene, (**C**) butane.

As mentioned in Section 2.2 Methods, the sorption properties were measured using TGA. The obtained data were used to calculate adsorption isobars of carbon dioxide, ethene, and butane. Isobars are presented in Figure 2, while the sorption properties at 303 K and under 1 atm are provided in Table 2. Adsorption isobars allow studying the sorption capacity for the particular gas of a particular material in some temperature ranges in just one measurement. However, the material needs to be fully regenerated at the maximum temperature of each cycle.

Based on the obtained isobars, it is possible to assess the sorption properties at the range of temperatures between 303 and 523 K for CO_2_ and C_2_H_4_ and between 303 and 523 K for butane (although regeneration is performed at 573 K). Typically, in the scientific literature, the TGA measurement is used for the characterization of sorbents and provides only one sorption capacity at one particular temperature. Moreover, this type of measurement performs the regeneration step in an inert atmosphere, which is followed by the adsorption step at the desired temperature, which might be further followed by another regeneration in an inert atmosphere. Changes of gases during thermogravimetric measurement lead to changes in the buoyancy effect. Using only one adsorbate during the measurement allows minimizing potential errors. Nevertheless, we have provided only values of gas update at 303 K, which should be easier for comparison for other researchers.

In Figure 2A, it is clearly visible that samples AC00, AC10, and AC30 should have a similar amount of crucial pores for CO_2_ adsorption. Material AC60 indicates a significant drop of CO_2_ uptakes due to the widening of narrow pores, which resulted in the highest volume of mesopores.

Moreover, the widening of narrow pores is additionally confirmed by C_2_H_4_ isobars. Material AC60, which showed a noticeable drop in CO_2_ uptake, indicated C_2_H_4_ uptake close to uptakes of AC10 and AC30. However, at higher temperatures, the sorption of ethene is clearly lower for material with the highest amount of mesopores than for other materials. On the other hand, material AC00 with the shortest time of activation has the lowest ethene uptake at 303 K, which is due to the relatively more developed microporous structure.

Figure 2C presents butane adsorption isobars. In this case, differences between isobars of particular materials are clearly visible. Butane uptake increases with the increasing mesopore volume; thus, the lowest and the highest uptakes were obtained for the AC00 (the lowest activation level) and AC60 (the highest activation level) materials, respectively.

Changes of sorption properties originate from different porous structures developed during the performed activation process. Other studies indicate that the crucial pores for adsorption (303 K, 1 atm) of CO_2_, C_2_H_4_, and C_4_H_10_ are 0.7 nm, 1.0 nm, and 2.5 nm, respectively. Based on this information, changes of the presented sorption properties might be investigated.

The activation time of 0 and 10 min resulted in the highest adsorption of carbon dioxide. This might suggest that samples AC00 and AC10 contain the highest amount of pores with diameters up to 0.7 nm. It needs to be noted that due to diffusion limitations, nitrogen adsorption at 77 K does not allow characterizing these pores. This might be done using CO_2_ adsorption at 273 K.

Longer activation time, i.e., 30 min, leads to a widening of narrow micropores. This results in the highest adsorption of ethene for material AC30; thus, it might be considered that AC30 contains the highest amount of pores up to a diameter of 1.0 nm. Widening of the pores results also in a slight decrease in CO_2_ uptake.

Physical activation performed for 60 min led to a further widening of pores, which resulted in the highest adsorption of butane. Therefore, material AC60 might have the highest amount of pores up to a diameter of 2.5 nm. Furthermore, the uptake of carbon dioxide, which is strongly influenced by narrow micropores, decreased noticeably, which is due to the further shifting of pore size distribution toward wider diameters. A similar tendency is observed for ethene uptake, although this decrease is small.

As presented above, the porosity of the material might be roughly estimated by the sorption capacities of particular gases with different molecular dimensions. However, knowledge about crucial pore diameters for specific gases is required.

Moreover, as it might be seen in Table 2, chemical activation provides the highest CO_2_ uptakes—3.7 and 3.8 mmol/g at 298 K. Physical activation does not provide such great results. However, it is much simpler, because it does not require pretreatment (impregnation with activating agent) and post-treatment (removal of activating agent). Moreover, carbon dioxide is a much safer chemical than KOH, which is typically used in chemical activation. On the other hand, materials obtained from beech wood compared to our previous studies [11,57] showed that it might be a competitive precursor in terms of the obtained sorption properties. An additional advantage is the high availability and relatively low price.

### 3.3. Structural Results

**Figure 3:**
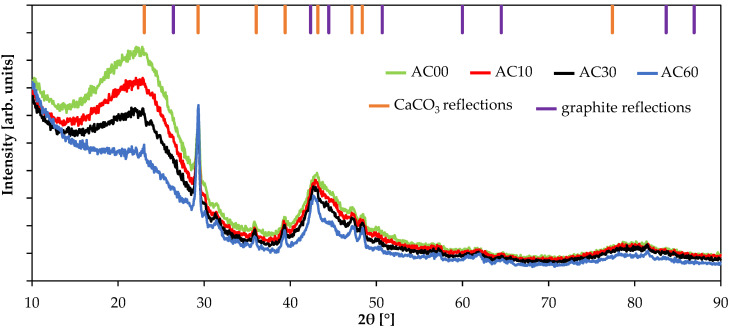
Diffractograms of activated carbons. Positions of reflections for CaCO_3_ (01-086-2340) and graphite (00-012-0212) were presented for reflections with relative intensity >10% and >2%, respectively.

Prepared materials were studied using the XRD technique. The obtained diffractograms are presented in Figure 3. These materials exhibit, typical for activated carbons, a turbostratic structure, which is clearly visible for material AC00. This structure indicates that the material contains very small regions of crystallographic order, but at a larger scale, the material might be considered amorphous. In case of activated carbon, crystallographic structures are just crystallites of graphite, although these crystallites tend to all have dimensions below 10 nm. Figure 3 contains the position of particular crystallographic planes, which can be distinguished in pure graphite material [11]. Moreover, during the activation, the carbon matrix is being removed through oxidizing (C + CO_2_ = 2CO). Thus, the inorganic impurities present in the raw material increase their content with increasing burn off (please refer to the following literature for diffractograms of activated carbons obtained from polymers without any impurities [11]). This study used beech wood that contained calcium, which after the activation procedure was present in the form of calcium carbonate (pattern 01-086-2340). Therefore, the diffractogram of the material AC60 with the highest activation level exhibits the highest content of CaCO_3_, as well as other inorganic impurities, which were not identified by the XRD technique.

Based on the obtained diffractograms, the following structural properties were calculated and presented in Table 3: stacking height (L_c_), interplanar distance (d), and average number of graphene planes in the graphite crystallites (N). All these properties are based on the (002) crystallographic plane. Furthermore, it is possible to calculate the lateral size of graphite crystallites (L_a_). However, it is very difficult for the presented results due to the presence of calcium carbonate, whose pattern almost overlaps with the crystallographic planes (100) and (101) of the graphite material. Moreover, calcium, as an element that is heavier than carbon, might even lead to a screening effect. Thus, the structural properties of material AC60 might be calculated with a higher error than other samples with a lower activation level.

The stacking height of graphite crystallites increases with the increasing activation level. It is a result of the removal of smaller crystallites by the reaction with carbon dioxide. When smaller crystallites are removed, the average value shifts toward higher values [11]. Moreover, the interplanar distance is decreasing, which might suggest that heteroatoms, mostly oxygen, are being removed from the crystallites. It is a well-known mechanism that during thermal treatment, functional groups decompose. In general, for carbonaceous materials, it is known as a graphitization process. Therefore, the interplanar distance tends to decrease toward 0.337 nm in pure graphite.

## 4. Conclusions

Based on the results presented in this article, beech wood might be considered as a suitable starting material for the production of activated carbon. Physical activation with carbon dioxide at 1273 K led to the sufficient development of a porous structure, while the time of the process affected the micro- or mesoporous characteristics of the obtained materials. After additional studies, the fine tuning of sorption properties could be possible.

The shortest activation time, i.e., instantaneous cooling after obtaining 1273 K, resulted in material with a high content of narrow micropores, which was proven by the highest CO_2_ uptake at 303 K. A longer activation time led to widening of the pores, decreasing and increasing uptakes of carbon dioxide and butane, respectively.

The activation process leads to the removal of the smallest graphite crystallites, which results in an increase in the crystallite’s average stacking height.

## Figures and Tables

**Table 1:** Textural properties of obtained materials. Results for materials from other studies were added for brief comparison.

Sample	Starting Material	SSA [m^2^/g]	Pore Volume [cm^3^/g]			Yield after Activation [%]	Ref.
			V_total_	V_micro_	V_meso_		
AC	Beech wood	182	0.08	0.06	0.02	-	This study
AC00	Beech wood	990	0.67	0.30	0.24	51	
AC10	Beech wood	1087	0.76	0.33	0.26	45	
AC30	Beech wood	1309	0.96	0.39	0.35	35	
AC60	Beech wood	1695	1.43	0.46	0.54	20	
AC	Eucalyptus wood	701	0.51	0.26	0.25	-	[48]
AC-H_3_PO_4_	Acacia wood	1039	0.55	0.34	0.18	46	[49]
P1:3-500	Chestnut wood	783	0.29	0.28	0.01	37	[39]
P55	Olive-tree wood	904	1.20	0.33	0.68	22	[50]
12	Coconut shell	1700	1.14	0.88	-	23	[51]
CSC-SALT-800	Cherry stones	1200	0.63	0.45	0.12	-	[52]
KJX-800-40-25.2	Bituminous coal	859	0.40	0.34	-	25	[53]
Urea 1:3:2	Peat	1100	0.87	0.31	0.56	20	[30]
940-5	PET	1830	-	0.60	0.01	41	[54]

**Table 2:** Sorption properties. Results for materials from other studies were added for brief comparison.

Sample	Starting Material	Sorption Properties, 1 atm [mmol/g]				Ref.
		CO_2_	C_2_H_4_	C_4_H_10_	Temperature	
AC	Beech wood	1.3	1.4	0.8	303 K	This study
AC00	Beech wood	2.1	2.9	2.6	303 K	
AC10	Beech wood	2.1	3.1	2.9	303 K	
AC30	Beech wood	2.0	3.3	3.8	303 K	
AC60	Beech wood	1.7	3.2	5.2	303 K	
H250-800	Palm fruit bunch	3.7	-	-	298 K	[55]
CACs-2-800	Coffee beans	3.8	-	-	298 K	[56]
AC30	Commercial kevlar	1.7	3.1	-	303 K	[57]
AC-20	PFA	1.8	2.1	-	303 K	[11]
Coal:ZnCl_2_	coal	-	-	1.9	303 K	[58]

**Table 3:** Structural properties of the obtained activated carbons.

Sample	Stacking Height Lc [nm]	d_(002)_ [nm]	N
AC00	1.07	0.393	2.72
AC10	1.03	0.393	2.62
AC30	1.08	0.392	2.76
AC60	1.22	0.391	3.12

## Data Availability

The data presented in this article will be available upon request.
